# Supplementary material for: Revealing pseudorotation and ring-opening reactions in colloidal organic molecules
Source: Nat Commun. 2021 May 14;12:2810. doi: 10.1038/s41467-021-23144-6 (PMC8121934; doi:10.1038/s41467-021-23144-6)
Supplement: Supplementary file 1 — Supplementary Information [file 41467_2021_23144_MOESM1_ESM.pdf]

Supplementary information for article: "Revealing pseudorotation and ring-opening reactions in colloidal organic molecules"

by Swinkels et al.

Supplementary Figures

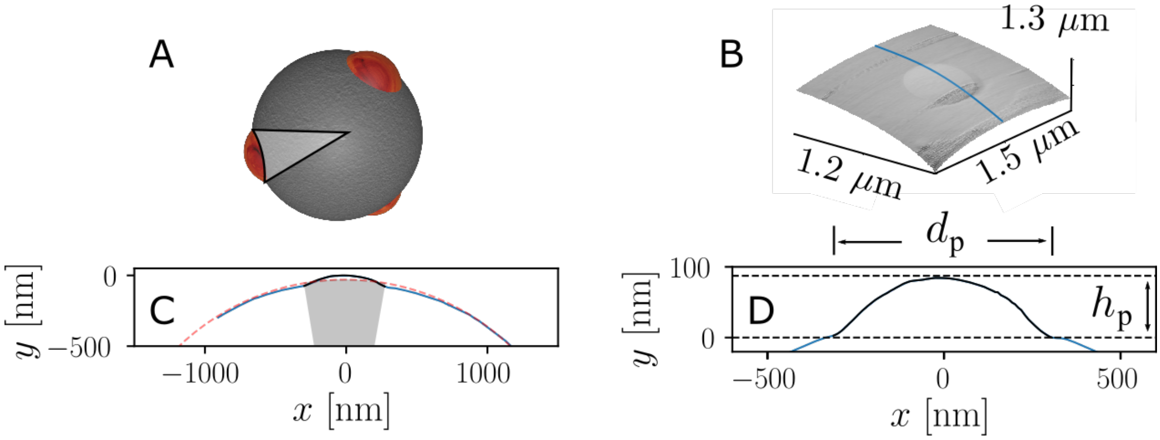

Supplementary Figure 1: **Atomic force microscopy (AFM) measurements of the tetrapatch particles.** (A) Schematic of tetrapatch particle, with the patch arc angle Θ_p indicated in white. (B) AFM image of a Tetrapatch particle. (C) 2D cuttrough of the AFM image along the blue line in panel (B). The blue solid line shows the bulk, the black solid line the patch. The dashed red line is the projected particle diameter σ. (D) Height profile zoomed in on patch, aspect ratio between x and y is set to 2. Patch diameter d_p, patch height h_p, and patch radius of curvature R_p can be determined from this image.

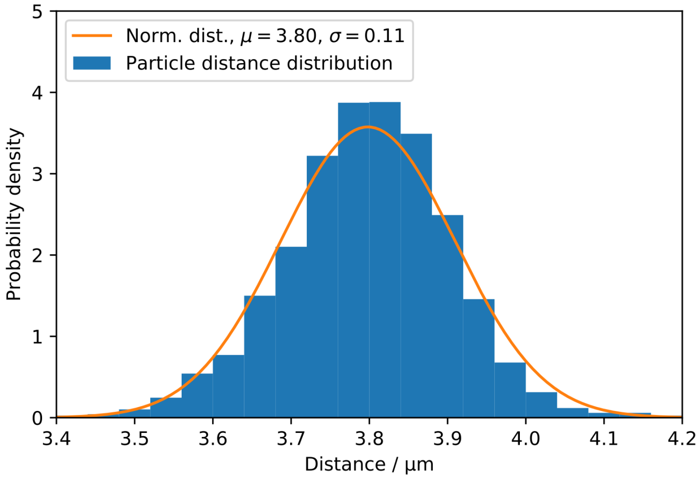

Supplementary Figure 2: **Distance between bonded tetrameric patchy particles.** Distribution of observed distances between tetrameric particles. In orange, we plot the normal distribution fit with mean distance between bonded particles $\mu = 3.80\,\mu\text{m}$ and standard deviation $\sigma = 0.11\,\mu\text{m}$. Source data are provided as a Source Data file.

Supplementary Tables

	σ [µm]	d_p [µm]	Θ_p [°]	h_p [nm]	R_p [µm]
tetrapatch	3.7(1)	0.54(5)	17(2)	84(5)	0.5(1)
dipatch	3.2(1)	0.58(5)	21(2)	45(5)	1.0(2)

Supplementary Table 1: The tetrapatch and dipatch particle diameter σ, projected patch diameter d_p, patch arc-angle Θ_p, patch height h_p, patch radius of curvature R_p are measured with AFM, an example is shown in Supplemantary Fig. 1. Source data are provided as a Source Data file.

Supplementary Notes

Supplementary Note 1: Optimizing the critical Casimir Force

We make use of critical Casimir forces to bind the patchy particles with either two patches (dipatch particles) or four patches (tetrapatch particles). The critical Casimir force arises in a near-critical binary solvent from the confinement of fluctuations between the particle surfaces, in analogy to the quantum mechanical Casimir force arising from the confinement of electromagnetic field fluctuations. Due to the universal relation between solvent correlation length and temperature, the critical Casimir effect offers universal temperature control over the particle attraction. In earlier work, we have shown that using temperature-dependent critical Casimir forces, we can reversibly assemble colloidal liquid and solid phases and study colloidal phase transitions [1, 2, 3]. Here, we apply this effect to achieve specific bonding of patchy particles. The advantage of the critical Casimir effect is that we can conveniently set the boundary conditions, i.e. the adsorption preference of the surfaces for one of the components of the binary mixture, by tuning the surface wetting properties. Nevertheless, in practice to achieve a high enough contrast between the wetting properties of the patch and bulk of a composite particle is challenging.

To optimize this contrast, we first investigated the assembly behaviour of the 2 particle components separately. We used the precursor PS spheres of the colloidal fusion synthesis, and polymerized TPM droplets under the same conditions as was used to polymerize the patches. This resulted in solid particles of diameter $d = 1\,\mu\text{m}$, that have the same surface properties as in the final composite patch particle.

We then vary the composition (lutidine concentration) of the binary solvent, and the salt concentration to determine the optimal selective TPM-TPM (and thus patch-to-patch) attraction. For this purpose, TPM and PS particles are dispersed in binary mixtures of varying lutidine concentration

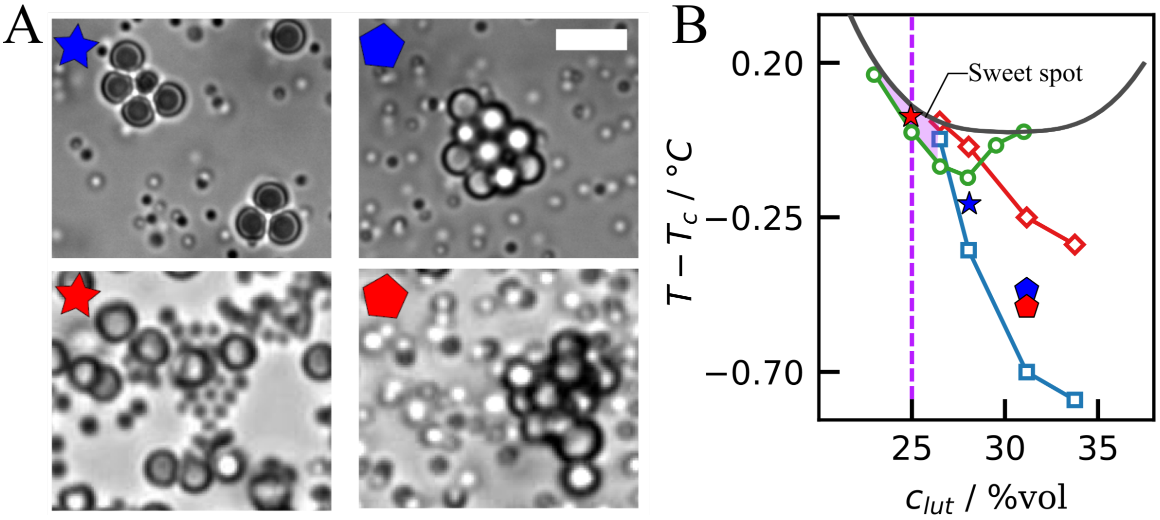

Supplementary Figure 3: **Enhancing the contrast for critical Casimir patch-to-patch binding** (A) Bright field images showing aggregation contrast of PS particles (big dots) and TPM particles (small dots). The former make the bulk, the latter the patch of the composite patchy particle. Clockwise, starting top left, conditions are: $c_{\text{lut}} = 28\%\text{vol}$, $[MgSO_4] = 0\,\text{mM}$; $c_{\text{lut}} = 32\%\text{vol}$, $[MgSO_4] = 0\,\text{mM}$; $c_{\text{lut}} = 25\%\text{vol}$, $[MgSO_4] = 0.5\,\text{mM}$; and $c_{\text{lut}} = 32\%\text{vol}$, $[MgSO_4] = 0.5\,\text{mM}$. Scalebar is $3\,\mu\text{m}$. (B) Aggregation diagram showing the shifted aggregation lines of TPM without salt (red diamonds), with $[MgSO_4] = 0.5\text{mM}$ (green circles), and PS without salt (blue squares). Black continuous line shows theoretical coexistence temperature of the binary mixture close to the critical point. Coloured stars and pentagons indicate corresponding measurement condition of (A). Purple dotted line indicates a good lutidine concentration for patchy assembly $c_{\text{lut}} = 0.25$. Source data are provided as a Source Data file.

for microscopic characterization. Samples were imaged while the temperature was slowly increased using a temperature-controlled stage. An oil-immersion objective with 63x magnification was used for imaging. By increasing the temperature slowly we identify the aggregation temperature T_{a}, at which clear cluster formation occurred (as shown in Supplementary Fig. 3a) and the coexistence temperature T_{cx}, at which bubbles form. From this, we can construct aggregation lines with respect to the solvent phase-separation line as a function of lutidine concentration, as shown in Supplementary Fig. 3b. The aggregation lines in Supplementary Fig. 3b show that without $MgSO_4$, PS particles (blue line) and TPM particles (red line) aggregate stronger for lutidine concentrations right of the critical composition, meaning that they both show the same water-philic affinity and thus no selective patch-to-patch attraction. However, by adding 0.5 mM of Magnesium Sulfate, the aggregation behaviour of TPM particles changes completely: a strong critical Casimir attraction is observed to the left of the critical composition, Supplementary Fig. 3b (green line). This indicates that the adsorption preference of TPM switches to lutidine-philic when adding $MgSO_4$, while the aggregation temperature of PS particles is little affected and remains water-philic as can be seen from the snapshots that show cluster formation of PS still happens at the right side of the critical temperature, Supplementary Fig. 3a (bottom right). Using the aggregation curves, we pinpoint a sweet spot where only TPM particles are expected to attract 3b (violet region).

We have also tested other salts, like KCl, and $CaCl_2$, but these did not yield the same selective TPM-TPM attraction. Though only partially understood, ions have been shown to effectively shift the adsorption preference of particles in binary mixtures, thereby strongly changing their critical Casimir interaction, see [4, 5, 6].

We perform all our experiments in this region, where we obtain selective patch-to-patch attraction: we use an optimal lutidine concentration $c_{\mathrm{lut}} = 25\%\mathrm{vol}$, with 1 mM $MgSO_4$ that shows the largest range of TPM-TPM (and thus path-to-patch) attraction while PS is not attractive.

Supplementary Note 2: Patchy Particle Assembly

We typically leave the particles to assemble at constant temperature for several hours. In Supplementary Fig. 4, we show the typical process of assembly in a sample with a mix of di- and tetra-patch particles, here at $\Delta T = 0.05$°C and number density $\rho = 0.015\,\mu\mathrm{m}^{-2}$. A bright-field microscope image of the assembled structures after 2 hours of assembly is shown in Supplementary Fig. 4A. A zoo of colloidal molecules is observed, from butanes and propanes to longer, polymer-like chains.

In Supplementary Fig. 4B we show a few snapshots revealing the assembly of colloidal methyl-cyclopentane. This process begins with butane and propane, which react to form hexane. This longer chain is now in a conformation in which it can easily form methyl-cyclopentane by reacting its first atom with the fifth atom in the chain. The molecule can also change to a conformation that reacts to cyclohexane by flipping atom 6 from the eclipsed to the staggered position. However, due to the quasi-2D nature of our system there is an energy penalty associated with this conformational change, making the formation of methyl-cyclopentane more likely.

Supplementary Fig. 4C and D show the growth of the mean cluster size and cluster size distributions. Initially, at the start of the measurement ($\Delta T = 0.26$°C), there is no clustering. As soon as we increase the temperature to $\Delta T = 0.05$°C, particles start assembling, and clusters start to grow. Supplementary Fig. 4C shows the growing average cluster size as a function of time. After 2 hours, when the measurement is stopped, an equilibrium cluster size has not been reached yet. The cluster mass distribution at different times is shown in Supplementary Fig. 4D. After 2 minutes

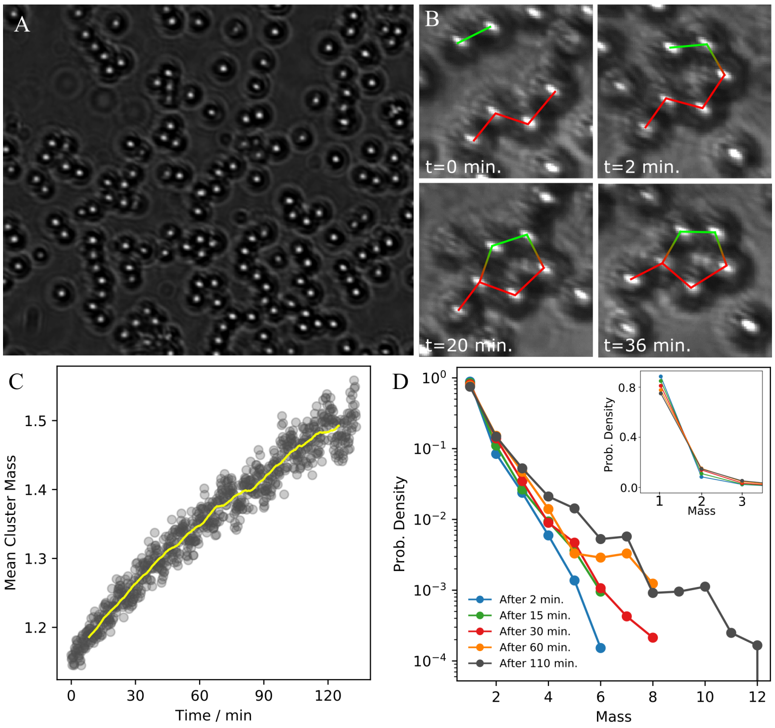

Supplementary Figure 4: **Patchy Particle Assembly.** We show a typical assembly experiment, where we set the temperature of the sample to $\Delta T = 0.05$°C. (A) Bright-field microscope image of tetra- and di-patch particles after two hours of assembly. The dimensions of the image are $97\,\mu\mathrm{m} \times 83\,\mu\mathrm{m}$. (B) Four bright-field microscope images showing the typical assembly of a colloidal (methyl-)cyclopentane molecule. In this particular case, we start with butane and ethane, which react to form hexane. The molecule then reacts with itself to form methyl-cyclobutane. (C) Growth of the average cluster mass with time. (D) Cluster mass distributions of the sample after 2, 10, 30, 60, and 110 minutes. The inset shows the first three data points plotted in a double-linear representation. Source data are provided as a Source Data file.

several structures have already formed. As time continues, the slope of the distribution becomes less steep, indicating large structures start forming, although singlets are still the major component. This behaviour is qualitatively in line with what we expect for a growing network according to Wertheim theory [7]. Also, it appears that at later times, structures with certain number n of particles are clearly preferred, such as $n = 5$, 7, and 10. We note that here, however, cyclic and linear structures are both counted; for a distinction of cyclic structures alone, see Fig. 1H of the main text.

Supplementary Note 3: Conformations of Different Colloidal Molecules

Apart from cyclopentane, which is treated quite thoroughly in the main text, we observe many other structures. To better understand the puckering of cyclic molecules, we investigated the bond angle distributions of a range of structures, from linear to cyclic compounds, as shown in Supplementary Fig. 5. We imaged propane, butane, cyclobutane and cyclopentane using bright-field microscopy in the same sample at constant temperature ($\Delta T = 0.05$°C), and measured their 2D-projected bond angles. From repeated fast imaging, we then determined bond angle distributions for all structures, as shown in the figure. We note that this projected 2D angle differs from the 3D angles explored in the main text, but does allow us to obtain significant statistics to compare the different linear and cyclic structures in terms of their bond stiffness.

Interestingly, propane and butane, both linear structures, one with three, one with four particles, show very similar angle distributions. Both species are free to move around their preferred angle: there is no steric hindrance or confinement effect from being in a ring. In fact, by treating the particle bond as a simple spring, we can determine a spring constant from the probability distributions in Supplementary Fig. 5. The unconstrained spring constant of propane and cyclobutane is $k_{\text{bend}} = 3.4 k_{\text{B}} T/rad$, shown as the solid black line. In contrast, the cyclic molecules (cyclobutane and cyclopentane) show much narrower angle distributions, which we associate with the constraint of the cyclic structure. The resulting "spring constant" of the cyclic structures is thus much stiffer than the one of the free bond angles of the linear structures.

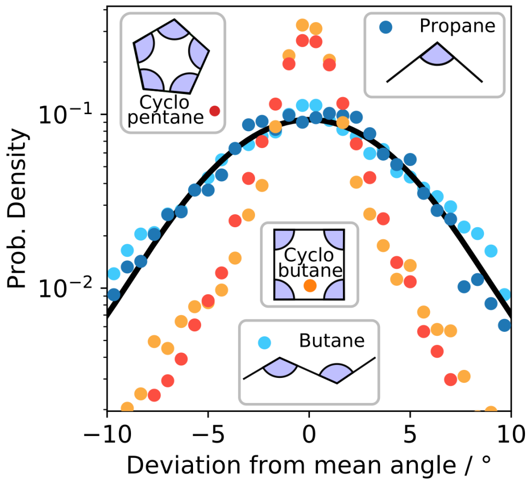

Supplementary Figure 5: **Bond stiffness of linear and cyclic colloidal molecules.** Distributions of the 2D-projected angles between the particles in different colloidal molecules: propane (navy), butane (turquoise), and cyclic compounds cyclobutane (orange) and cyclopentane (red). Note that the x-axis shows the deviation from the mean angle, $\theta_{\text{deviation}} = \theta - \langle\theta\rangle$. The insets show which angles we used to build the angle distributions shown here. The black solid line shows a Hookean fit of the angles with a spring constant of $k_{\text{bend}} = 3.4 k_{\text{B}} T \text{rad}^{-1}$. Source data are provided as a Source Data file.

Supplementary Note 4: Timescales

To probe the typical timescale of the ring transitioning between conformations, we acquired bright-field images of rings at a frame rate of 2 s^{-1}. In each frame, we track the particles in a horizontal plane and determine the angles θ between the projected particle positions. We take these time-dependent angles to calculate the time autocorrelation.

$$g_2(\Delta t) = \frac{\langle \theta_i(t) \times \theta_i(t+\Delta t)\rangle_{i,t}}{\langle \theta_i(t)^2\rangle_{i,t}} \tag{1}$$

where we average over all five angles and the observation time interval $\Delta t = 0.5$ seconds. We plot a typical example of the autocorrelation of the angles over time in Supplementary Fig. 6. Generally, we find there is a fast component, τ_1, of around 9 seconds, and a slower component, τ_2, of around 25 seconds. We associate τ_1 with the diffusion of the particles, as this is approximately the expected timescale for a 3.7 µm particle to diffuse its own radius. Furthermore, we associate τ_2 with the configurational changes, i.e. pseudo-rotation of the ring, which is further confirmed by the anticorrelated motion of neighbouring particles, indicating the characteristic change between conformations while keeping the overall puckered state (see main text).

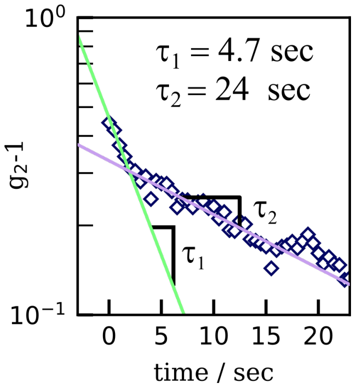

Supplementary Figure 6: **Timescale of dynamics.** Autocorrelation function of the inter-particle angles of the 2D projection of colloidal cyclopentane as a function of time in half-logarithmic representation (see Supplementary Eq. 1). Two characteristic timescales are observed: a fast one, of around 4.7 seconds, and a slower timescale of around 24 seconds. Source data are provided as a Source Data file.

Supplementary Note 5: Simulation of the structural distribution of colloidal cyclopentane

The structural distribution of colloidal cyclopentane is sampled with the computational model based on critical Casimir potentials [8] benchmarked onto experimentally measured chain length distributions and the bending rigidities of a dipatch particle system at various temperatures.

Here the computational model for tetrapatch particles interacting via critical Casimir interactions is presented at an off-critical binary mixture and the simulations of the conformational distribution of colloidal cyclopentane is discussed.

The potential of the tetrapatch particle

The potential of the patchy particle system (Supplementary Eq. 2) has two contributions: the pair potential V_{pair} (Supplementary Eq. 3) acting between the colloidal particles and a gravitational potential V_{gravity}. The total interaction is a summation over the pair potentials and the gravity over N particles.

$$V = \sum_{i,j}^{N} V_{\text{pair}}(i,j) + \sum_{i}^{N} V_{\text{gravity}}(z_i) \tag{2}$$

$$V_{\text{pair}}(i,j) = \begin{cases} \infty, & D = r - \sigma \leq 0 \\ V_{\text{rep}}(D_{ij}) + V_{\text{C}}(D_{ij}) S'(\Theta_i) S'(\Theta_j), & D > 0 \end{cases} \tag{3}$$

The pair potential is composed out of an isotropic repulsion V_{rep} plus a critical Casimir attraction V_{C} only effective at the patches via a switching function S' of both particles i and j. The repulsion and attraction are only a function of the radial surface-surface distance D_{ij} between two particles with diameter σ and center-to-center distance r, while the switching function S' is dependent on the patch orientation with respect to the interparticle vector.

In experiment, the tetrapatch particles and dipatch particles are synthesized from the same materials and assembled into various architectures in the same solvent: a water/lutidine (75/25%vol) binary liquid with 1.0 mM $MgSO_4$. Only the physical dimensions of the dipatch and tetrapatch particles differ as shown in Supplemantary Table . Thus, one expects that the potential of the

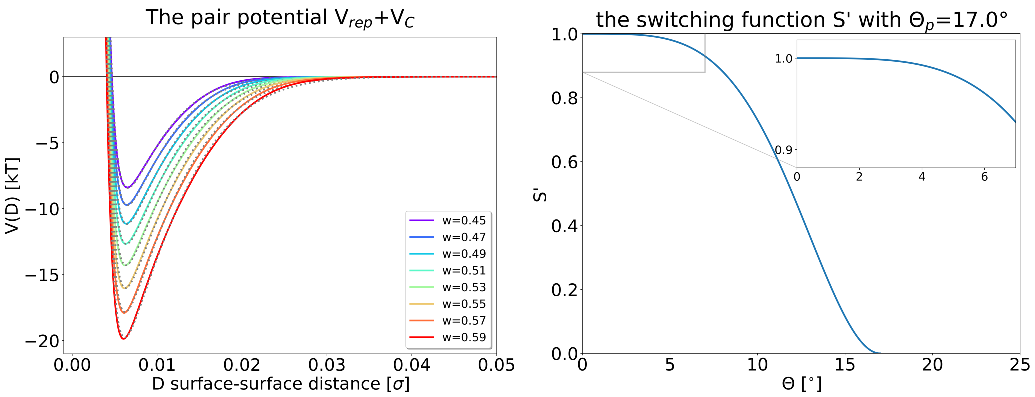

Supplementary Figure 7: **Pair potential and switching function.** On the left, pair potential of a hydrophobic isotropic particle with a radius of 0.5 μm, immersed in a water/lutidine (75%vol/25%vol) solution with 1.0 mM $MgSO_4$ is illustrated at various wetting scaling parameters w(color) with the fitted functions (grey dotted line). On the right, the switching functions S' (Eqn 4) of a particle with Θ_p=17.0°.

tetrapatch particles uses the benchmarked parameters of the dipatch system, i.e. surface charge $\Upsilon = -0.10\,e\,\mathrm{nm}^{-2}$ and scaling wetting parameter $w = 0.47$, which are material properties.

However, significant bonding of the tetrapatch particles is only observed at temperatures of $\mathrm{d}T = T_{\mathrm{cx}} - T \leq 0.04$ where T_{cx} is the phase separation temperature. This indicates that upon increasing the temperature and approaching $\mathrm{d}T = 0.04$ in experiment, the potential gets stronger and the critical temperature T_{c} has not been crossed yet. This is in contrast to the theoretically predicted critical temperature at $\mathrm{d}T = 0.08$*. The addition of salt to the solvent may affect the phase separation curve which leads to a shift of T_{cx}.

With the non-universal scaling constant B=0.765 and wetting scaling parameter $w = 0.47$, patches of tetrapatch particles do not show significant binding at $\mathrm{d}T = 0.04$ [9]. Therefore, w is increased to ensure spontaneous formation of bonds. From an interaction strength with $w \geq 0.55$, there are colloidal cyclopentanes observed in an MC simulation at 15%area coverage including gravity. Supplementary Fig. 7 on the left shows the pair potentials and their fit at various scaling wetting parameters w of a hydrophobic isotropic colloidal particle with $\Upsilon = -0.10\,e\,\mathrm{nm}^{-2}$, $R_p = 0.5\,\mu\mathrm{m}$, and $\mathrm{d}T = 0.04\,\mathrm{K}$ immersed in the binary liquid.

The angular dependence of the interaction strength is captured by the switching function S which is a smoothly decaying function from 1 to 0 as shown in Supplementary Fig. 7 on the right. As the patch geometry of the tetrapatch is similar to the particles from Ref [10], we use the switching function

$$S'(\Theta_i) = \begin{cases} \frac{1}{2}\left[1 - \cos\left(\frac{\pi(\cos\Theta_i - \cos\Theta_{\mathrm{p}})}{1-\cos\Theta_{\mathrm{p}}}\right)\right] & \cos\Theta_i \geq \cos\Theta_{\mathrm{p}} \\ 0 & \cos\Theta_i < \cos\Theta_{\mathrm{p}} \end{cases} \tag{4}$$

where Θ_{p} is the patch arc-angle and $\cos\Theta_i = \mathbf{r_{ij}} \cdot \mathbf{p}_i / |\mathbf{r}_{ij} \cdot \mathbf{p}_i|$ is the angle between the patch vector $\mathbf{p}_i$ and the interparticle vector $\mathbf{r}_{ij}$.

The gravitational potential is a function of the mass difference between the colloidal particle and the solvent. Tetrapatch particles are synthesized from four equal spheres of polystyrene (PS) surrounding one sphere of TPM with an ideal ratio $r_{\mathrm{TPM}}{:}r_{\mathrm{PS}} = (\sqrt{8} - 2)/2$ which translates into a fractional volume $\phi_{\mathrm{PS}} = 1 - \phi_{\mathrm{TPM}} \approx 0.9825$.

Monte Carlo sampling of the colloidal cyclopentane conformations

The computational system is composed of N=5 particles in a cyclopentane conformation. We sample the conformational distribution of the colloidal cyclopentane by performing 60.000 cycles of 500.000 single particle Monte Carlo steps. Bond breakage, if the critical Casimir interaction $E_{\mathrm{C}} = 0$, is not allowed as we are only interested in the conformational distribution of cyclopentane. Additionally, to mimic the experimental measurement accuracy, we add a Gaussian noise with zero mean and standard deviation of $0.1\,\mu\mathrm{m} = 0.03\sigma$ in the direction perpendicular to the wall.

The conformational distribution of the colloidal cyclopentane

Supplementary Fig. 8 shows the probability density of colloidal cyclopentane with the potential with $w = 0.55$ as function of puckering phase ϕ and amplitude q_{N} of system with and without gravity and the Gaussian noise. As observed in experiment, there is no preference for the envelope or twist conformation in any of the simulations. Thus, the puckering phase is not affected by the gravitational force acting on the colloidal particles and the measurement inaccuracy in the z-direction.

The puckering amplitude does depend on the presence of gravity and the measurement inaccuracy in the z-direction. Without gravity the maximum of the probability density $q_{\mathrm{N,max}}$ is shifted toward larger q_{N} values of approx. 0.15 compared to the experimental measurement. This means that the colloidal cyclopentane is more bend in a system without gravity. With gravity the colloidal

*The location of T_{cx} with respect to T_{c} calculated via $\frac{T_{\mathrm{cx}} - T_{\mathrm{c}} T_{\mathrm{x}}}{=} \left(\frac{|c_{\mathrm{c}} - c|}{B}\right)^{1/\beta}$ where $T_{\mathrm{c}} = 33.86\,^{\circ}\mathrm{C}$ is the critical temperature, $c_{\mathrm{c}} = 0.287$ the critical (lutidine mass) fraction, $B = 0.765$ a non-universal scaling constant of the water lutidine solution without salt measured in Ref. [9], and $\beta = 0.3265$ a universal scaling constant.

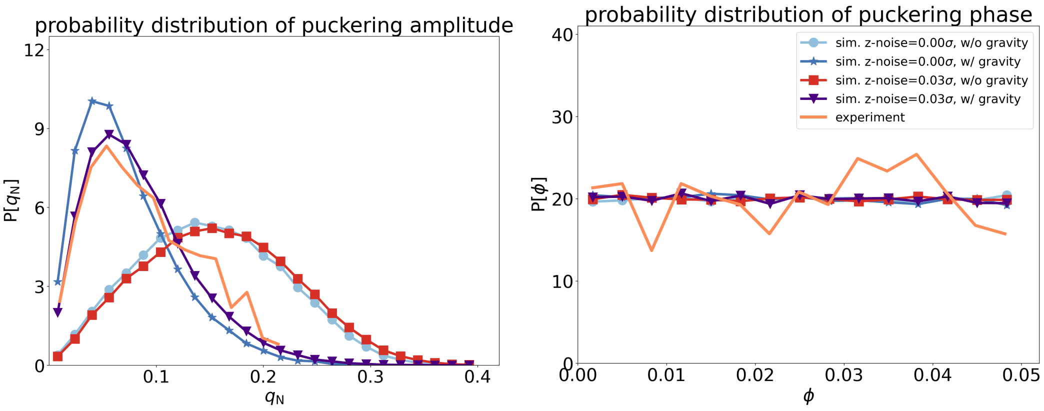

Supplementary Figure 8: **Puckering amplitude and phase in experiments and simulations.** Probability distributions of puckering amplitude q_{N} (left) and phase ϕ (right) in simulation and experiment. The simulations are conducted with and without adding Gaussian noise with a standard deviation of 0.03σ in the z-direction and gravity. The colour coding is equal in both graphs. Source data are provided as a Source Data file.

cyclopentane is flattened and the maximum of the probability density is close to experiment $q_{\mathrm{N}} = 0.06$. The effect of the Gaussian noise is making the colloidal cyclopentane appear more bend than it actually does. This effect is mainly observed in the system without gravity. An increase of density at q_{N} values larger that $q_{\mathrm{N,max}}$ is observed which shift the complete curve.

Supplementary Note 6: The Bending Energy of Cylopentane

In the main text, we combine our experimental observations of the puckering ring with simulations for the entropic component to extract the pure bending energy of the ring as a function of q_{N}. To do this, we start with the free energy as a function of puckering amplitude q, given by

$$F(q) = U(q) - TS(q) \tag{5}$$

where U is the Boltzmann averaged energy caused by bending, T the temperature, and S the entropy. Assuming this free energy to exhibit a Boltzmann distribution in thermal equilibrium, the probability of observing a configuration with puckering amplitude q is given by

$$f(q) \sim e^{-F/k_{\mathrm{B}}T} = e^{-U/k_{\mathrm{B}}T} \cdot e^{TS/k_{\mathrm{B}}T} \tag{6}$$

Rewriting this gives:

$$\begin{aligned} ln(f(q)) &= - U(q)/k_{\mathrm{B}}T + S(q)/k_{\mathrm{B}} + C \\ U(q)/k_{\mathrm{B}}T &= - ln(f(q)) + S(q)/k_{\mathrm{B}} + C \end{aligned} \tag{7}$$

with C a constant. The entropic contribution is given by the multiplicity Ω as determined from the simulations of random independent displacements.

$$S(q) = k_B \cdot ln(\Omega(q)) \tag{8}$$

$$U(q)/k_BT = ln(\Omega(q)) - ln(f(q)) + C \tag{9}$$

$$\begin{aligned} &= ln(\Omega(q)/f(q)) + C \\ &= ln(P_0 \cdot P(q)/f(q)) + C \\ &= ln(P(q)/f(q)) + C' \end{aligned} \tag{10}$$

with Ω the number of microstates, and $P(q)$ the entropic simulation observation frequency.

Supplemantary Methods

Supplemantary Methods 1: Colloidal Catalytic Conversion

To study the catalytic conversion of colloidal cyclopentane, we bring a ring into contact with a hydrophobic surface. To do this, we treat our sample capillaries with a gas silanization reaction. The capillaries are cleaned thoroughly using a piranha treatment. Capillaries are then placed in a vacuum desiccator together with approx. 1 ml of hexamethyldisilazane (HMDS) ($\geq$ 99.0%, Sigma-Aldrich). Pressure is lowered to below 200 mbar using a pump. Pressure is kept low for at least 2 hours. The capillaries are then baked in an oven at 120 °C for circa 1 hour.

Samples are then prepared as usual in these capillaries. In these experiments, we use smaller patchy particles (diameter = 1.8 µm, preparation analogues to description given in the method section of the main text) because they sediment slower, and have a larger gap between T_{a} and T_{cx} of 0.20 °C. Particles are left to sediment on one side of the capillaries. Then, we raise the temperature to $\Delta T = 0.10$ °C and flip the sample over so the particles are in free fall. In this free fall, the particles assemble into (among other things) cyclopentane. When the ring is almost at the bottom of the sample, we lower the temperature to $\Delta T = 0.17$ °C, and observe the adsorption of the ring to the surface and its subsequent breakup using a 100x oil-immersion objective.

Supplemantary Methods 2: Details of Particle Tracking

As was mentioned in the main text, we image the assembled structures using confocal microscope image stacks, alternating with bright field images. To follow a colloidal molecule in time, we acquire around 100 image stacks and bright field images during a time interval of 12 minutes. The bright field images are processed using particle tracking software (Trackpy, see [11]) to determine the centre of the patchy particle in the horizontal plane. The 3D locations of the fluorescent features are determined using the same software.

Since each particle has a set of 4 fluorescent patches, we must now determine which set of four patches is part of the same particle. We first find the centres of particles from the bright field channel. We go through these particles one by one, and determine which features in the confocal channel are within the particle diameter d of the bright field particle. Then, within this group of features, we determine all inter-feature distances and angles. Based on these parameters, we can judge which 4 patches are part of the same particle, and which features are due to a nearby neighbouring particle(s) or other error(s). Taking the centre of mass of the patches yields an accurate 3D position vector $\mathbf{R}_j$ of each particle j of a ring.

Finally, we use an algorithm which eliminates all rings which have unrealistically high or low angles between particles to further eliminate any data with particles placed on the wrong positions, which we then confirm by manual inspection of the video

Supplementary References

[1] Nguyen, V. D., Faber, S., Hu, Z., Wegdam, G. H. & Schall, P. Controlling colloidal phase transitions with critical Casimir forces. *Nature Communications* **4**, 1584 (2013).

[2] Dang, M. T., Verde, A. V., Nguyen, V. D., Bolhuis, P. G. & Schall, P. Temperature-sensitive colloidal phase behavior induced by critical Casimir forces. *The Journal of Chemical Physics* **139**, 094903 (2013).

[3] Nguyen, T. A. *et al.* Switching colloidal superstructures by critical casimir forces. *Advanced Materials* **29**, 1–6 (2017).

[4] van Duijneveldt, J. S. & Beysens, D. Adsorption on colloids and flocculation: The influence of salt. *J. Chem. Phys.* **94**, 5222–5225 (1991).

[5] Pousaneh, F., Ciach, A. & Maciołek, A. How ions in solution can change the sign of the critical Casimir potential. *Soft Matter* **10**, 470–483 (2014).

[6] Nellen, U. *et al.* Salt-induced changes of colloidal interactions in critical mixtures. *Soft Matter* **7**, 5360–5364 (2011).

[7] Sciortino, F., Bianchi, E., Douglas, J. F. & Tartaglia, P. Self-assembly of patchy particles into polymer chains: A parameter-free comparison between Wertheim theory and Monte Carlo simulation. *Journal of Chemical Physics* **126** (2007).

[8] G. Stuij, S., Labbé-Laurent, M., E. Kodger, T., Maciołek, A. & Schall, P. Critical Casimir interactions between colloids around the critical point of binary solvents. *Soft Matter* **13**, 5233–5249 (2017).

[9] Mohry, T. F., MacIoek, A. & Dietrich, S. Phase behavior of colloidal suspensions with critical solvents in terms of effective interactions. *Journal of Chemical Physics* **136** (2012).

[10] Guo, R., Mao, J., Xie, X. M. & Yan, L. T. Predictive supracolloidal helices from patchy particles. *Scientific Reports* **4**, 1–7 (2014).

[11] Allan, D., Keim, N., Caswell, T., van der Wel, C. & Trackpy community. Trackpy v0.3.0. Zenodo (2014).
