# Supplementary material for: Revealing pseudorotation and ring-opening reactions in colloidal organic molecules
Source: Nat Commun. 2021 May 14;12:2810. doi: 10.1038/s41467-021-23144-6 (PMC8121934; doi:10.1038/s41467-021-23144-6)
Supplement: Supplementary file 3 — Description of Additional Supplementary Files [file 41467_2021_23144_MOESM3_ESM.docx]

**Description of Additional Supplementary Files**

**Supplementary Movie 1: Puckering of colloidal cyclopentane 1**

Reconstructed movies showing the real-space puckering motion of colloidal cyclopentane from four different perspectives: along the x, y and z directions, as well as from an inclined direction to help facilitate 3D viewing. The raw bright field and confocal microscope images are also included.

**Supplementary Movie 2: Puckering of colloidal cyclopentane 2**

Another example of a reconstructed movie showing the real-space puckering motion of colloidal cyclopentane from four different perspectives, including the bright field and confocal microscope images.

**Supplementary Movie 3: Puckering motion through phase space 1**

Movie showing the puckering motion of colloidal cyclopentane in real space, together with the corresponding movement in q-ϕ space.

**Supplementary Movie 4: Puckering motion through phase space 2**

Another example showing the puckering motion of colloidal cyclopentane in real space, together with the corresponding movement in q-ϕ space.

**Supplementary Movie 5: Ring opening reaction**

Reconstructed movies showing the ring-opening reaction of colloidal cyclopentane from four different perspectives: from the x, y, z directions and from an inclined direction. The ring opening reaction occurs at the end of the movie, at a particle strongly puckered out of plane. The raw bright field and confocal microscope images are also included.
